# Dual Function of CD81 in Influenza Virus Uncoating and Budding

**DOI:** 10.1371/journal.ppat.1003701

**Published:** 2013-10-10

**Authors:** Jiang He, Eileen Sun, Miriam V. Bujny, Doory Kim, Michael W. Davidson, Xiaowei Zhuang

**Affiliations:** 1 Department of Molecular and Cellular Biology, Harvard University, Cambridge, Massachusetts, United States of America; 2 Department of Chemistry and Chemical Biology, Harvard University, Cambridge, Massachusetts, United States of America; 3 Program in Virology, Harvard Medical School, Harvard University, Boston, Massachusetts, United States of America; 4 National High Magnetic Field Laboratory and Department of Biological Science, The Florida State University, Tallahassee, Florida, United States of America; 5 Department of Physics, Harvard University, Cambridge, Massachusetts, United States of America; 6 Howard Hughes Medical Institute, Cambridge, Massachusetts, United States of America; Imperial College London, United Kingdom

## Abstract

As an obligatory pathogen, influenza virus co-opts host cell machinery to harbor infection and to produce progeny viruses. In order to characterize the virus-host cell interactions, several genome-wide siRNA screens and proteomic analyses have been performed recently to identify host factors involved in influenza virus infection. CD81 has emerged as one of the top candidates in two siRNA screens and one proteomic study. The exact role played by CD81 in influenza infection, however, has not been elucidated thus far. In this work, we examined the effect of CD81 depletion on the major steps of the influenza infection. We found that CD81 primarily affected virus infection at two stages: viral uncoating during entry and virus budding. CD81 marked a specific endosomal population and about half of the fused influenza virus particles underwent fusion within the CD81-positive endosomes. Depletion of CD81 resulted in a substantial defect in viral fusion and infection. During virus assembly, CD81 was recruited to virus budding site on the plasma membrane, and in particular, to specific sub-viral locations. For spherical and slightly elongated influenza virus, CD81 was localized at both the growing tip and the budding neck of the progeny viruses. CD81 knockdown led to a budding defect and resulted in elongated budding virions with a higher propensity to remain attached to the plasma membrane. Progeny virus production was markedly reduced in CD81-knockdown cells even when the uncoating defect was compensated. In filamentous virus, CD81 was distributed at multiple sites along the viral filament. Taken together, these results demonstrate important roles of CD81 in both entry and budding stages of the influenza infection cycle.

## Author Summary

As a “Trojan Horse” that only encodes 13 viral proteins, influenza hijacks host cell machinery for productive infection. In this work, we studied the role of the host protein CD81 in influenza infection. We found that CD81 was important for influenza infection at two distinct stages: virus uncoating and virus budding. Specifically, during virus entry, more than half of internalized virus particles were trafficked into a specific CD81-positive endosomal population for virus uncoating. Depleting CD81 led to a significant defect in viral uncoating and infection. During virus egress, CD81 was recruited to virus assembly site, and incorporated into individual virions at specific sub-viral locations. CD81 depletion resulted in virions that failed to detach from the plasma membrane and a marked decrease in progeny virus production.

## Introduction

Influenza virus, the major causal agent of flu, is an enveloped, negative-sense RNA virus containing three viral membrane proteins: hemagglutinin (HA), neuraminidase (NA), and M2 proton channel. Encapsulated within the viral envelope is a layer of matrix protein (M1) and a segmented genome. The eight single-stranded RNAs package into viral ribonucleoprotein complexes (vRNPs), each attached to a RNA-dependent RNA polymerase complex with three subunits: PA, PB1, and PB2 [1].

As an obligatory pathogen that encodes only 13 viral proteins, influenza virus must rely on host proteins and cellular machinery to complete its infection cycle. Influenza infection begins with virus binding to sialic acids on the plasma membrane [2]. Virus-receptor interaction subsequently triggers viral entry through multiple endocytic routes including clathrin-mediated endocytosis, a clathrin/caveolin-independent pathway, and macropinocytosis [3]–[7]. Upon internalization, virus particles are trafficked from early endosomes to maturing endosomes, where fusion between the virus and endosomal membranes results in release of vRNPs into the cytoplasm followed by nuclear import of the vRNPs [1], [8]–[10]. As replication proceeds, viral mRNAs are exported out of the nucleus for protein translation, and viral components are trafficked to the plasma membrane, the site of virus assembly and progeny virion egress [11].

Recently, several genome-wide siRNA screens identified host factors exploited by influenza virus [12]–[16]. CD81 emerged as a top candidate in two screens and was found to regulate early viral entry steps [15], [16]. CD81 belongs to the family of tetraspanins and is expressed on both plasma and endosomal membranes [17]–[19]. It associates with other tetraspanins and tetraspanin-interacting proteins to form tetraspanin-enriched microdomains [18], [20], [21]. Together, these proteins regulate many cellular processes such as cell adhesion, cell signaling, cell migration, and protein trafficking [18], [19], [22]–[24]. Tetraspanins are known to play an important role in different steps of viral infection [25]. For example, CD81 functions as a co-receptor for hepatitis C virus (HCV) [26]–[29]. CD81 interacts with HCV glycoprotein E2 to prime the virus for low-pH dependent fusion during entry [26], [30], [31]. In addition to mediating viral entry, CD81 is also potentially involved in viral assembly. CD81 is one of the cell-derived components incorporated into purified influenza virus particles [32]. It is however unknown how CD81 facilitates influenza viral entry, or whether CD81 plays a functional role in influenza virus assembly.

We conducted a comprehensive study from viral entry to egress to examine the effect of CD81 depletion on influenza infection. Upon dissecting each of the major steps in influenza infection pathway, we found that CD81 was required for productive viral infection and that CD81 primarily functions at two stages: viral fusion within endosomes and virus budding. About half of the influenza virus particles that fused in cells underwent fusion within CD81+ endosomes, and CD81 depletion led to a decrease in virus fusion and infection. CD81 was highly enriched in the virus budding zones and recruited to specific sub-viral locations. During virus assembly, CD81 initially formed small clusters at the growing tip of assembling virus, and then localized at both the growing tip and budding neck of spherical or slightly elongated virions. CD81 depletion led to an increase in the propensity of budding virions to remain attached to the plasma membrane and a reduction in progeny virus production. These findings demonstrate a dual function of CD81 in both entry and budding of influenza viruses.

## Results

### CD81 is involved in both early and late stages of influenza virus infection

**Figure 1:**
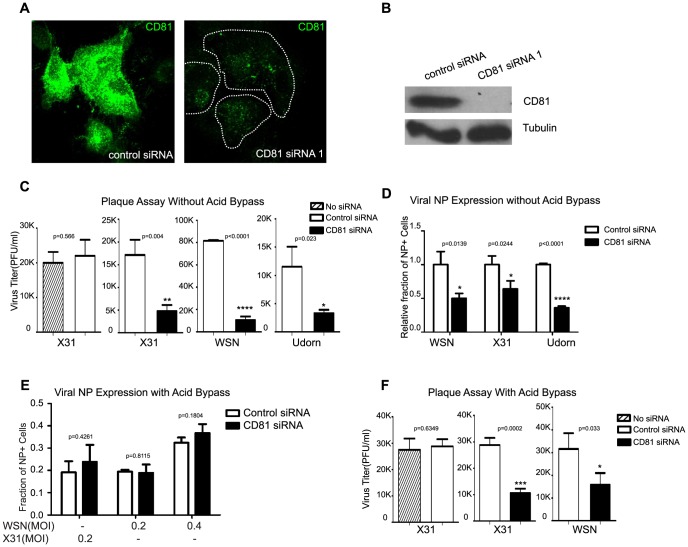
CD81 is involved in both early and late stages of influenza virus infection. A) A549 cells were treated with non-targeting control siRNA, or CD81 siRNA1 for 48 hours and immunostained with anti-CD81 antibody. Images are maximum projections of confocal z-stacks. B) A549 cells were treated with control or CD81 siRNA for 48 hours. Cells were harvested for western blotting with indicated antibodies. Tubulin was used as a loading control. C) Influenza virus infection is impaired by CD81 depletion. A549 cells were treated with siRNAs or mock treated for 48 hours and subsequently infected with X-31, WSN, or Udorn at a MOI of <0.1 for 36 hours. The viral titer in the supernatant was determined by plaque assays. The shaded bar indicates the infectivity measured in cells mock treated with a transfection solution that contains no siRNA; the hollow bars indicated the infectivity measured in cells treated with control, non-targeting siRNA; the black solid bars indicate the infectivity measured in cells treated with CD81 siRNA. D) The number of infected cells expressing viral NP is reduced by about 50% upon CD81- knockdown. siRNA-treated A549 cells were infected with WSN, X-31, or Udorn viruses with a MOI of <0.1 for 8 hours without acid bypass and the fraction of cells expressing NP was measured through flow cytometry. E) Viral NP expression is unaffected upon CD81 knockdown when influenza infection is induced by the acid-bypass treatment to eliminate the entry defect. siRNA-treated A549 cells were allowed to bind with WSN or X-31 virus on ice for 1 hour, treated with warm low pH PBS buffer (pH 4.5) for 2 minutes. After 8 hours, cells were collected and stained against NP for flow cytometry analysis. F) Virus titer in the supernatant is reduced by ∼50% or more in CD81-knockdown cells infected by influenza viruses through the acid-bypass treatment. Briefly virus was allowed to bind with control or CD81-knockdown cells on ice for 1 hour. Unbound virus particles were washed out and low pH buffer was added in for 2 minutes to trigger virus fusion at the plasma membrane. At 17 hours post infection, supernatant was collected and the viral titer was assayed by plaque assay. For figure 1C–1F, the error bars are standard deviation derived from three independent experiments. A two-tailed student *t-test* was performed for all of the numerical data, and the p value of the data is shown. A p value smaller than 0.05 indicates there is a statistically significant difference.

To elucidate the role of CD81 in influenza virus infection, we used CD81 knockdown by siRNA to probe the effect on three different influenza A virus strains: influenza A/WSN/33 (H1N1), a lab-adapted strain that mainly produces spherical virus particles; influenza A X-31, A/Aichi/68 (H3N2) which has a slightly elongated shape [33]; and influenza A/Udorn/72 (H3N2), which can produce long filamentous virus [34]–[37]. We screened six CD81 siRNA constructs, including several previously reported ones [15], [16], and found that CD81 siRNA 1 gave the highest knockdown efficiency (Figure 1A, B and Figure S1A, B). After 48 hours, siRNA 1 yielded 80–85% CD81 knockdown (Figure S1C). Treatment with siRNA 1 specifically depleted CD81, whereas the expression levels of several known CD81-associating proteins, including CD9, CD82, CD63, integrin β1 (ITGB1), EGFR, and EWIF, were not affected (Figure S1D). In subsequent experiments, we used siRNA 1 to deplete CD81 in A549 lung carcinoma cells.

To assay the effect of CD81 depletion on the production of infectious viral progeny, siRNA-treated A549 cells were infected with WSN, X-31 or Udorn virus at a MOI of <0.1 for 36 hours. The virus titer of the supernatant was determined using plaque assays. Compared to the non-targeting control siRNA treated cells, the CD81-knockdown cells exhibited a substantial decrease in virus titer: ∼90% decrease for WSN, ∼75% decrease for X-31, and ∼70% decrease for Udorn (Figure 1C). These results are consistent with the previously published data [15], [16], and indicate that multiple different influenza strains require CD81 for infection.

Next, we proceeded to determine which stage(s) of the multi-step influenza-infection process is CD81-dependent. To test whether CD81 affects early infection, we infected siRNA-treated A549 cells and measured the expression of NP, the first viral protein expressed in influenza-infected cells [15], [16], [38]. We assayed the fraction of cells that express NP (NP+) as well as the level of NP expression in each NP+ cell using flow cytometry. For all three viral strains, CD81-knockdown cells had ∼50% fewer NP+ cells, as compared to non-targeting siRNA treated control cells (Figure 1D). Among the NP+ cells, the NP expression level was similar between control and CD81-knockdown cells (data not shown). These results suggest that CD81 is involved in early infection either at the step of or prior to viral protein expression.

In order to test whether CD81 directly affects viral protein expression, we next induced viral fusion (uncoating) at the plasma membrane through an acid-bypass treatment: treatment with a buffer of pH below 5, the pH value required for HA-induced membrane fusion. We then probed the expression of NP in these samples. The fraction of NP+ cells and the level of NP expression were similar between control and CD81-knockdown cells infected by the virus using the acid-bypass treatment (Figure 1E and data not shown). These results suggest that CD81 is not directly involved in the viral protein expression, and the inhibition of virus infection by CD81 knockdown was most likely due to inhibition of viral uncoating in endosomes or any step prior to uncoating. It is worth noting that this acid-bypass assay overcame the entry defect for the WSN and X-31 strain, but a similar acid bypass treatment did not work for the Udorn strain, likely because low pH causes fragmentation of filamentous influenza viruses [7].

To probe whether CD81 plays additional roles beyond viral uncoating, we induced influenza viral fusion at the plasma membrane using the acid-bypass treatment to overcome the entry defect, and determined the virus titer of the supernatant 17 hours post-infection. Notably, as compared to control siRNA treated cells, the virus titer was decreased by more than 50% in the CD81-knockdown cells (Figure 1F). This defect did not result from a decrease in viral gene expression, as the percent of viral protein expressing cells and the expression level at 17 hours post-infection were unaffected upon CD81 knockdown (Figure 1E and data shown later). These results suggest that CD81 affects another step post viral gene expression.

Taken together, the above results indicate that CD81 is important for two distinct stages of the influenza infection cycle: one during the early infection at or prior to viral uncoating and one during late infection after viral gene expression. In the following experiments, we aimed to identify the specific roles of CD81 in influenza virus infection.

### CD81 is not involved in virus binding, internalization or trafficking to early endosomes

**Figure 2:**
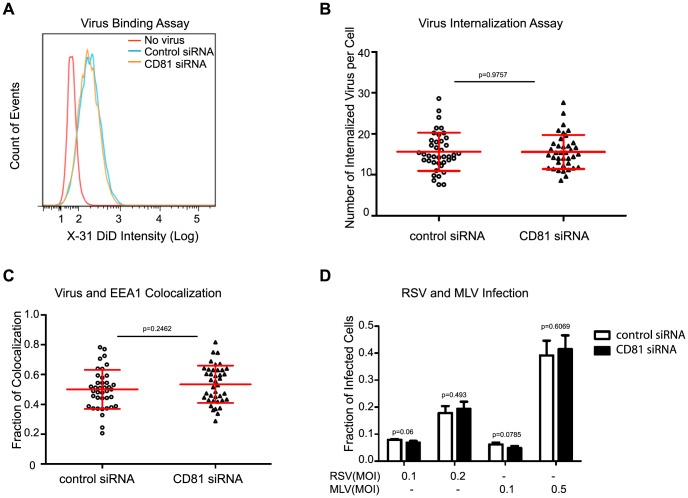
CD81 is not required for virus binding, internalization or delivery into early endosomes. A) CD81-knockdown does not affect virus binding, as measured by flow cytometry. The magenta, blue and orange curves correspond to the intensity profiles measured for cells without adding viruses, control cells after influenza virus binding, and CD81-knockdown cells after influenza virus binding, respectively. B) The number of virus particles internalized is not affected by CD81 knockdown. The number of internalized virus particles was shown in a dot plot, with the middle line representing the mean value, and top/bottom line representing standard deviation. At least 40 randomly chosen cells were analyzed for each condition. C) The percent of virus particles colocalizing with early endosome is not affected by CD81 knockdown. Early endosomes were immunostained with anti-EEA1 antibody. Data was plotted similarly as in (B). At least 40 randomly chosen cells were analyzed for each condition. D) CD81 depletion does not affect RSV or pseudo-typed MLV infection. siRNA-treated A549 cells were infected with different doses of RSV and pseudo-typed MLV virus for 24 hours. For RSV virus infection, RSV fusion protein expression was quantified by flow cytometry, while for pseudo-typed MLV virus, the GFP signal was analyzed. A two-tailed student *t-test* was performed for all of the numerical data, and the p value of the data is shown.

To identify the CD81-dependent entry step(s), we conducted a series of experiments to examine the effect of CD81 on virus binding, internalization, transport into early endosomes, and fusion. siRNA-treated A549 cells were first incubated with fluorescently labeled X-31 virus for 30 minutes at 4°C, a temperature that inhibits endocytosis. The amount of surface-bound virus was analyzed through flow cytometry. CD81-knockdown cells exhibited no defect in binding with influenza viruses when compared to control cells treated by non-targeting siRNA (Figure 2A). Next, influenza virus infection was allowed to proceed for 30 minutes at 37°C, and the number of internalized virus particles was quantified. As shown in Figure 2B, the number of internalized viruses per cell was similar between control and CD81-knockdown cells. Moreover, virus particles in both control and CD81-knockdown cells were delivered into early endosomes after internalization at 37°C. At 20 minutes post infection, the mean fraction of virus particles colocalized with early endosomes (marked by EEA1) was ∼50% and ∼53% for control and CD81-knockdown cells, respectively (Figure 2C). These results suggest that CD81 does not affect trafficking of influenza virus to early endosomes.

Additionally, we tested the effect of CD81 depletion on two other viruses: respiratory syncytial virus (RSV) and GFP-encoding pseudotyped murine leukaemia virus (MLV), which are known to undergo virus fusion in early endosomes in a pH-independent manner [39], [40]. Pseudotyped MLV infected cells express GFP, but cannot produce complete virions, allowing the quantification of MLV entry through measuring the GFP expression. For RSV, we measured the expression level of the fusion protein (F protein) after 24 hours of infection. As shown in Figure 2D, the fraction of infected cells was similar between control and CD81-knockdown cells with MLV and RSV infection. This data further corroborates the notion that CD81 does not affect virus trafficking into early endosomes.

Taken together, the results presented above indicate that CD81 is not involved in influenza virus binding, internalization, or trafficking into early endosomes.

### Influenza virus particles are delivered to and fuse in CD81+ endosomes

**Figure 3:**
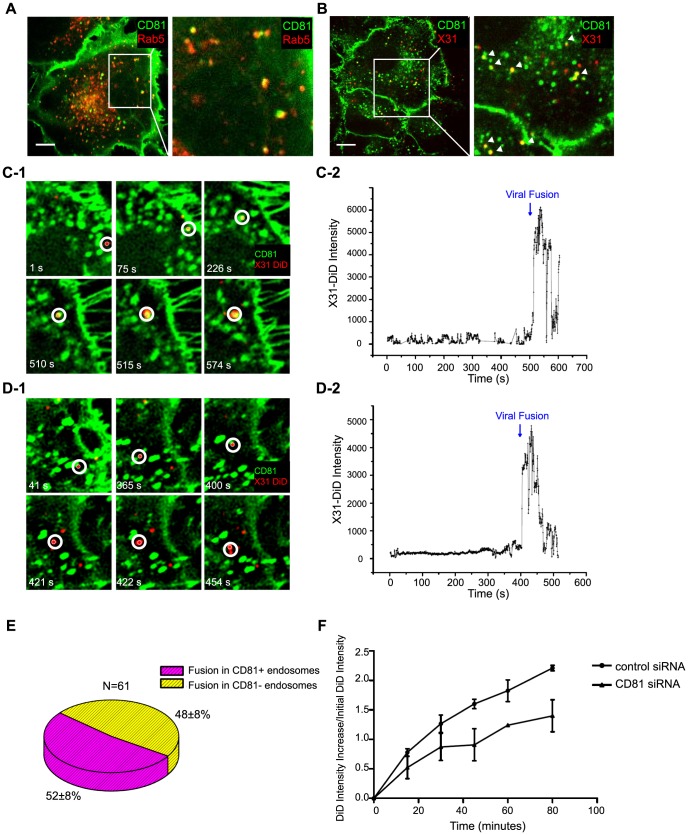
A major fraction of viruses are trafficked to and fuse in CD81-positive endosomes. A) CD81 substantially colocalizes with Rab5. A549 cells were electroporated with CD81-mEmerald and RFP-Rab5. At 24 hours, the cells were fixed and imaged. An enlarged image of the boxed region is shown on the right. Scale bar: 10 µm. B) Influenza virus particles traffick into CD81+ endosomes. A549 cells were cold bound with Alexa Fluor 647-labeled X-31 virus (red) on ice for 30 minutes and then chased for 15 minutes at 37°C. The samples were fixed and immunostained against CD81 (green). An enlarged image of the boxed region is shown on the right. All of the images are confocal XY cross sections. Scale bar: 10 µm. C) An influenza virus particle enters and fuses within a CD81-positive endosome after entry. Live-cell confocal imaging of DiD-labeled X-31 added *in situ* to CD81-mEmearld expressing A549 cells maintained at 37°C. The images were collected with a 0.5 s interval. C-1) Several snapshots taken at different time points with the virus indicated by the white circles. C-2) The fluorescence signal of the indicated DiD-labeled virus as a function of time. Note that there is a sudden increase of DiD signal at 515 s, which indicates a viral fusion event. D) Influenza virus can also fuse in a CD81-negative endosome. D-1) Several snapshots taken at different time points with the virus indicated by the white circles. D-2) The fluorescence signal of the indicated DiD labeled virus as a function of time. The virus particle fused at 422 s. E) Among 61 virus particles tracked from binding to fusion, 52±8% enter and fuse within CD81+ endosomes whereas the remaining 48±8% fuse in CD81- endosomes. The results are taken for four independent experiments, and the ±error indicates the standard deviation derived from these experiments. F) Virus fusion is impaired upon CD81 depletion. DiD-labeled X-31 was allowed to bind with A549 cells on ice for 30 minutes, and then chased for the indicated times at 37°C. Cells were trypsinized and fixed immediately, and analyzed by flow cytometry. The increase in the DiD intensity versus the initial DiD intensity is plotted. The error bars are standard deviation derived from duplicate experiments.

Next, we probed the role of CD81 in virus fusion. Influenza virus is trafficked from early endosomes to maturing endosomes, in which the low pH environment triggers conformational changes in HA that mediate viral fusion with the endosomal membrane [2], [9], [41]. In addition to being distributed on the plasma membrane, CD81 also showed substantial colocalization with early and maturing endosomes, which are Rab5 positive (Rab5+) (Figure 3A) [41]. About 30–35% Rab5+ endosomes contained CD81 (Figure 3A), suggesting that CD81 is enriched in a sub-population of these endosomes. To probe whether influenza virus particles are delivered into CD81+ endosomes, we allowed Alexa Fluor 647-labeled X-31 to internalize for 15 minutes and immunostained the cells for CD81. As shown in Figure 3B, a substantial fraction (∼54%) of virus colocalized with CD81+ endosomes.

We then tracked individual influenza virus particles in living cells, a technique that has been previously established [5], [10], [41]–[43], to examine whether influenza viruses fuse in CD81+ endosomes. To this end, we expressed CD81-mEmerald in A549 cells. Similar to endogenous CD81, CD81-mEmerald was localized on both plasma and endosomal membranes (Figure S2A) and the expression of CD81-mEmerald did not affect the fraction of endosomes that are CD81+ (Figure S2B–D). Moreover, the expression of CD81 also did not affect influenza viral fusion or infectivity (Figure S2E, F).

To facilitate tracking of individual virus particles, X-31 viruses were labeled with a saturating amount of DiD, a lipophilic dye, such that the fluorescence emission from the DiD molecules was low due to a self-quenching effect between neighboring dyes. Fusion between the virus envelope and endosomal membrane should lead to an increase in fluorescent intensity (dequenching), due to the diffusion of dyes from the virus into the lipid bilayer of the endosomes [42]. We added labeled viruses to the CD81-mEmerald expressing cells *in situ* at 37°C. The virus particles typically show restricted movement immediately after binding to the cell, followed by rapid and directed movement once the virus particles are internalized, similar to our previous observations [5], [41], [42]. We observed a proportion of virus particles entering into CD81+ endosomes soon after internalization, as illustrated by the example shown in Figure 3C and movie S1. These viruses remained colocalized with CD81 and eventually fused with the CD81+ endosomes, as reflected by the sudden increase of DiD fluorescence, presumably after the endosomes matured to acquire a sufficiently low pH (Figure 3C and movie S1). Among the 61 virus particles that we tracked from binding to fusion, about 52±8% underwent viral fusion within CD81+ endosomes (Figure 3E). The remaining 48±8% of virus particles fused in endosomes lacking CD81 (Figures 3D,E, and movie S2). To confirm that the fusion events indeed occurred in endosomes, we tracked individual DiD-labeled influenza virus particles in cells expressing RFP-Rab5. Similar to previous observations [41], nearly 90% of the viral fusion events occurred in Rab5+ endosomes (Figure S3).

To investigate whether CD81 affects viral fusion, we next monitored the DiD fluorescent intensity in control and CD81-knockdown cells that were infected with DiD-labeled X-31 virus. In these experiments, cells were first incubated with DiD-labeled virus at 4°C and then the temperature was increased to 37°C to initiate viral entry. At specific time points after the temperature shift, infected cells were collected and the DiD fluorescence from these cells was quantified with flow cytometry. As expected, there was a consistent increase of DiD fluorescence with time due to viral fusion (Figure 3F). Notably, compared to control cells, CD81-knockdown cells exhibited a significant reduction in viral fusion (Figure 3F), suggesting that CD81 facilitates viral fusion. Most of the remaining viral fusion events in CD81-knockdown cells still occurred in Rab5+ endosomes (Figure S3). The reduction in viral fusion was, however, incomplete (Figure 3F), consistent with the observation that only about half of the virus particles fuse in CD81+ endosomes (Figure 3E), though the incomplete inhibition of viral fusion could also be in part due to the incomplete knockdown of CD81 (Figure S1).

These data indicate that CD81 marks a specific population of Rab5+ endosomes that are responsible for half of viral fusion events. Because CD81-knockdown cells reduced viral infection (Figure 1C) and exhibited a higher reduction in virus titer when infected without acid bypass than when infected with acid bypass (Figures 1C,F), viral fusion within CD81+ endosomes likely leads to productive influenza infection.

Taken together, our results indicate that half of virus particles are trafficked to and undergo viral fusion in CD81+ endosomes. CD81 could facilitate viral fusion by organizing endosomal membrane to assist viral fusion or helping virus traffick to fusion-competent endosomal compartments.

### CD81 is not involved in the expression or trafficking of viral proteins

**Figure 4:**
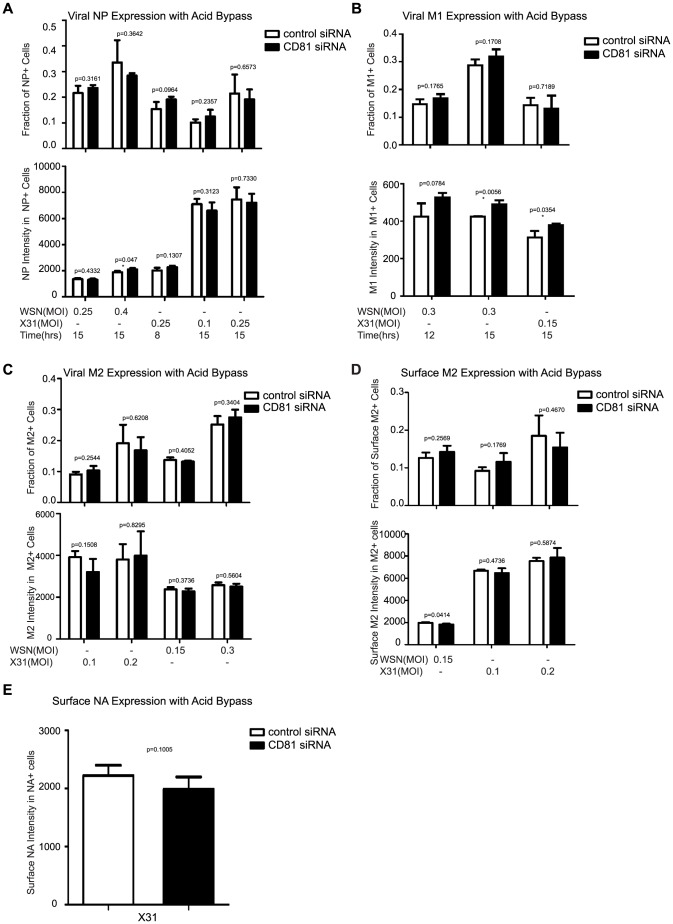
CD81 depletion does not affect viral protein expression and transport. A) CD81 knockdown does not affect the expression of viral NP protein in cells infected by influenza viruses with the acid-bypass treatment. Experiments were performed similarly as in 1E) except that the expression levels are evaluated at different time point post infection and with different dose of viruses. The percent of NP-expression cells and the NP expression level in NP+ cells are plotted. B) CD81 knockdown does not affect the expression of viral M1 protein in cells infected by influenza viruses with the acid-bypass treatment. Experiments were performed similarly as in (A) except cells were immunostained for M1. The percent of M1+ cells and the M1 expression level in the M1+ cells are plotted. C) CD81 knockdown does not affect the expression of viral M2 protein in cells infected by influenza viruses with the acid-bypass treatment. Experiments were performed similarly as in (A) except cells were immunostained for M2. The percent of M2+ cells and the M2 expression level in the M2+ cells are plotted. D) CD81 knockdown does not affect the amount of M2 protein trafficked to the cell surface in cells infected by influenza viruses with the acid-bypass treatment. Experiments were performed similarly as in (C) except cells were stained for M2 without permeabilization. The percent of M2+ cells and the surface M2 expression level in M2+ cells are plotted. E) CD81 knockdown does not affect the amount of NA protein trafficked to the cell surface in cells infected by influenza viruses with the acid-bypass treatment. The NA expression level was estimated from confocal images in control or CD81 siRNA treated cells infected by X-31virus. A two-tailed student *t-test* was performed for all of the numerical data, and the p value of the data is shown.

In the subsequent experiments, we aimed to determine which post-entry step(s) of the viral infection process are CD81 dependent. To this end, we examined the effect of CD81 on viral protein expression, viral protein trafficking, and the assembly and egress of progeny viruses. Our initial results in Figure 1E suggested that CD81 knockdown did not directly affect viral NP expression. This was further substantiated by infecting cells with various viral doses across different time points using the acid-bypass treatment. As shown in Figure 4A, the fraction of NP+ cells and NP expression level increased with the viral dose and infection time, while there was no significant difference between control and CD81-knockdown cells. To further validate the finding, we measured the expression of another cytosolic viral protein, M1. Similar to the results on NP, CD81-knockdown cells infected by influenza viruses using the acid-bypass treatment did not exhibit a difference in the fraction of M1+ cells or the level of M1 protein expression, when compared to the control cells (Figure 4B). These data suggest that CD81 does not play a role in viral gene expression for cytosolic viral proteins.

To determine whether CD81 affects viral membrane protein expression, we probed M2 expression with acid bypass treatment. The fraction of M2+ cells and the M2 expression level in M2+ cells were similar between control and CD81-knockdown cells (Figure 4C). Furthermore, by only probing the surface M2 protein without permeabilizing the cells, we found that there was no difference in surface M2 protein expression level either (Figure 4D), indicating that CD81 knockdown also did not affect the trafficking of M2 to the cell surface. Similarly, the expression and trafficking of another viral membrane protein, NA, were also not affected upon CD81 depletion (Figure 4E).

Taken together, these data indicate that CD81 does not play a direct role in the expression of influenza viral proteins or the trafficking of influenza membrane proteins to the plasma membrane.

### CD81 is recruited to influenza virus budding sites on the plasma membrane

**Figure 5:**
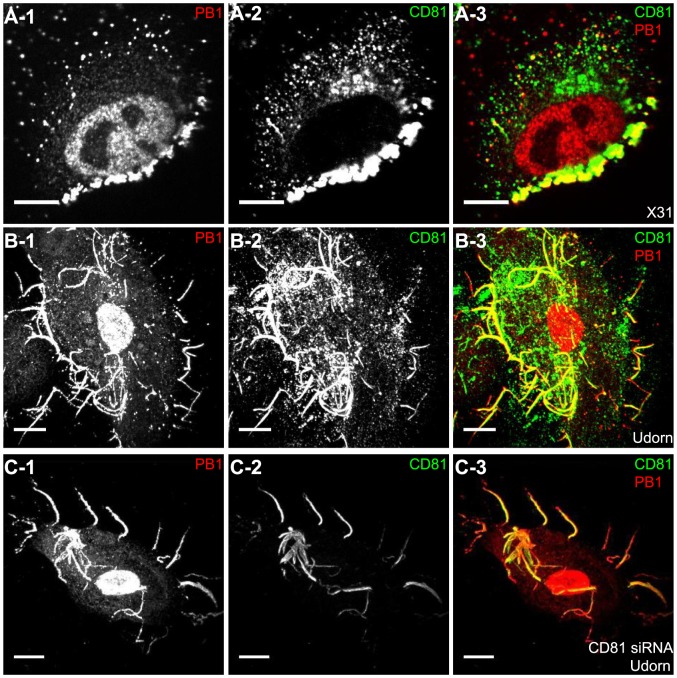
CD81 is recruited to the virus budding sites. A) CD81 is recruited to the virus budding zone in X-31 infected cells. A549 cells were infected with X-31 for 16 hours. Cells were stained with anti-CD81 antibody (green) and anti-PB1 antibody (red). Images are confocal XY cross-sections. Scale bar: 10 µm. B) CD81 is incorporated into budding filamentous virions of Udorn-infected cells. A549 cells were infected with Udorn virus for 16 hours, and stained with anti-CD81 antibody and anti-PB1 antibody. Scale bar: 10 µm. C) Remaining CD81 in CD81-knockdown cells is incorporated into budding filamentous viruses of Udorn infected cells. Similar to (B) except that CD81-knockdown cells were used. The CD81 expression level in Udorn-infected cells was calculated based on confocal images of more than 100 cells, and was found to be decreased by ∼88% upon CD81 depletion as compared to control cells. The amount of CD81 per viral filament was reduced by 63% compared to that in untreated cells. Scale bar: 10 µm.

Next, we probed the role of CD81 in virus assembly. To test whether CD81 is present at the viral assembly sites, A549 cells were infected with the three influenza strains and immunostained for CD81 and the viral protein. Notably, with X-31 infection, CD81 was mostly localized to a site concentrated with multiple viral proteins (Figures 5A and S4A, B). In contrast to uninfected cells, which showed a uniform distribution of CD81 on the plasma membrane, X-31-infected cells exhibited marked redistribution of CD81 into concentrated patches. All of the X-31 proteins that we could obtain specific immunofluorescence staining for, including PB1, NA and M2, were present in these patches. The CD81 patches were formed on the plasma membrane, as confirmed by immunofluorescence of non-permeabilized cells (Figure S4B). We note that there was only a modest decrease of CD81 expression upon viral infection (Figure S5A, B). For Udorn-infected cells, CD81 was enriched along the budding virus filaments marked by PB1 (Figure 5B). PB1 is a good filament marker that colocalized with Udorn HA and M2 in the budding filamentous virions (Figure S4C,D). We have also directly observed colocalization between CD81 and other Udorn proteins including HA, NA and an anti-Udorn serum (Figure S4E–G). Remarkably, upon siRNA treatment, which depleted 80∼85% of the endogenous CD81, the remaining CD81 was all concentrated in the budding viral filaments, whereas the cell body had little CD81 signal (Figures 5C and S4H). The average amount of CD81 per virus filament in the CD81-knockdown cells was reduced by more than 60% compared to that in control cells. Although our lack of WSN antibodies made it difficult to perform similar immunofluorescence experiments on WSN-infected cells, our EM images with CD81 labeled by immunogold showed that CD81 was also recruited to the WSN virus budding zones (Figure S6A). Taken together, these results indicate that CD81 is specifically recruited to the influenza virus assembly and budding sites.

To probe which viral component may be responsible for recruiting CD81, we turned to a plasmid-based system that expresses only specific viral envelope proteins in cells [44]. We transiently transfected the plasmid containing HA or NA in A549 cells and immunostained the cells with CD81 and HA or NA. Interestingly, HA tends to form clusters on the cell surface even when expressed alone in A549 cells and CD81 accumulated substantially in the HA clusters (Figure S7A). About 46% of HA clusters colocalized with CD81. In contrast, NA when expressed alone did not form clusters but was distributed largely uniformly across the plasma membrane and there was no appreciable correlation between the CD81 distribution and NA distribution (Figure S7C). Mock transfection with plasmid that did not contain HA or NA did not yield any appreciable HA or NA staining (Figure S7B,D). These results suggest that HA is likely responsible for recruiting CD81 to the viral budding sites.

### CD81 is incorporated at specific sub-viral locations and facilitates influenza virus budding

**Figure 6:**
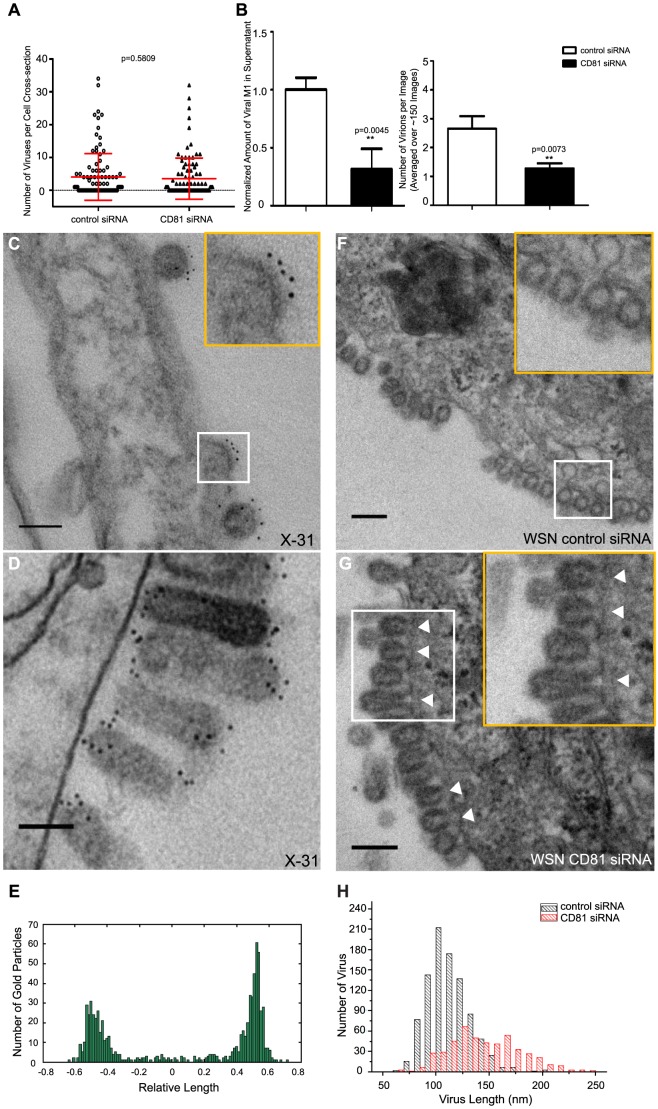
CD81 is enriched at specific sub-viral sites of budding virions and CD81 knockdown impairs virus scission. A) CD81 knockdown does not change the number of budding virions attached to infected cells. siRNA-treated cells were infected with WSN virus with the acid-bypass treatment for 15 hours. Cells were directly fixed for transmission electron microscopy and the number of budding virus particles per cell cross-section is quantified for over 250 sections, and presented in the dot plot. A two-tailed student *t-test* was performed and the p value is provided. B) CD81 knockdown causes a substantial reduction in the number of released virus particles. siRNA-treated cells were infected with WSN virus with the acid-bypass treatment for 17 hours. The amount of viral M1 protein in the supernatant was probed with ELISA. The number of M1 positive and HA positive virus particles in the supernatant was counted using immunofluorescence imaging. The error bar is standard deviation from three independent measurements. C) CD81 localizes at the tip of growing X-31 viruses during the early budding stages. Cells were infected with X-31 for 12 hours and CD81 was immunogold labeled for electron microscopy. An enlarged image of the area in the white box is shown in the upper right corner. Scale bar: 100 nm. D) CD81 mainly localizes at the tip and budding neck of the X-31 viruses during late budding stages. Similar to (C) except the infection time was 16 hours. Scale bar: 200 nm. E) Distribution of gold particles in budding X-31 viruses at 16 hour post infection. To align the virus particles, the length of each virus is normalized to 1, with its middle point assigned with coordinate value of 0. For individual gold particles on the budding virus, their coordinate values were calculated based on their relative distance to the middle point. Coordinates with negative values correspond to positions close to the plasma membrane. A total of 105 budding viruses were analyzed. F) Budding WSN viruses exhibit a spherical morphology with fully enclosed membrane envelope in control siRNA-treated cells. A549 cells were infected with virus with the acid-bypass treatment for 13 hours. The region in the white box is magnified and shown in the upper right corner. Scale bar: 200 nm. G) Budding WSN viruses are more elongated in CD81 siRNA treated A549 cells. A substantial fraction of budding viruses have an open membrane neck connected to the plasma membrane (indicated by arrowheads). The region in the white box is magnified and shown in the upper right corner. Scale bar: 200 nm. H) Budding WSN viruses are elongated upon CD81 depletion, as shown by the distribution of budding virus length in control or CD81-knockdown cells.

Although we observed a ∼50% or more decrease in virus titer in CD81-knockdown cells after acid-bypass treatment to overcome the CD81-dependent entry defects (Figure 1F), it remained unknown whether the defect in viral titer stems from a decrease in the number of budding virions assembled on the cell, the number of progeny virus particles released from the cell, or the specific infectivity per released virus particle. To distinguish between these possibilities, we first infected cells using the acid-bypass treatment and then quantified the number of budding virions attached to the cells using transmission electron microscopy. After quantifying more than 250 cell cross-sections per condition, we performed statistical analysis and found no statistically significant difference in the number of assembling virus particles per cell cross-section in the control versus CD81-knockdown cells (Figure 6A).

Next, we infected cells with WSN using the acid-bypass treatment, collected the virus particles in the supernatant, and then quantified the amount of viral matrix protein M1 using an ELISA assay and the number of released virus particles positive of both M1 and HA using an imaging assay (Figure 6B). Notably, compared to control cells treated by non-targeting siRNA, CD81-knockdown cells exhibited 50% or more decrease in both the amount of viral M1 and the number of M1+ and HA+ virus particles released into the supernatant. These results suggest that the CD81-knockdown-induced reduction in viral titer in cells infected by the acid-bypass treatment stems from a defect in virus release. Given that the reduction in viral titer (Figure 1F) was similar to the reduction in the amount of released viral proteins or viral particles (Figure 6B), we did not further probe the change in specific infectivity per virus particle.

To examine how CD81 may facilitate release of progeny virus particles, we next probed the distribution of CD81 within individual budding virions using immunogold electron microscopy. In X-31-infected cells, CD81 was readily observed in budding virions (Figure 6C, D). During early assembly stages, CD81 clusters located at the growing tip of budding virions (Figure 6C). Interestingly, when viruses grew into a mature, slightly elongated shape [33], CD81 was not only found on the growing tip, but also on the neck of budding virions (Figure 6D). The elongated morphology of X-31 allowed us to quantitatively analyze the CD81 distribution in virions by aligning the long axis of the virus particles and normalizing the position of immunogold-labeled CD81 to the total length of the virus. Remarkably, CD81 is highly enriched at the two ends of the budding virions (Figure 6D, E).

Similarly, we also found CD81 to be enriched in budding WSN virus particles, but the quantity of immunogold detected per WSN virus is substantially lower than that in X-31 viruses, which made it difficult to determine the CD81 distribution in these viruses (Figure S6A). However, the nearly perfectly spherical shape of the WSN virus allowed us to detect an interesting morphological defect of budding viruses in CD81-knockdown cells. When we examined budding virions in cells infected by WSN, we found that most budding WSN viruses were spherical and completely enclosed by viral envelope in control cells treated by non-targeting siRNA (Figure 6F). In stark contrast, budding WSN virus in CD81-knockdown cells appeared much more elongated (Figure 6G). The average length of budding virions in control and CD81-knockdown cells was ∼100 nm and ∼150 nm, respectively (Figure 6H). Furthermore, many budding viruses in CD81-knockdown cells did not have a fully enclosed envelope but remain attached to the plasma membrane through an open membrane neck (indicated by arrowheads in Figure 6G). We performed similar experiments with the X-31 strain. Again, we consistently observed many budding X-31 virions with the open budding neck defect upon CD81 depletion (Figure S6B, C), though characterizing whether the budding virions were further elongated was difficult due to the large variation of the virion length of the pleomorphic X-31.

Taken together, the specific enrichment of CD81 at the neck of the budding virions, the defect in budding neck closure in CD81-knockdown cells, and the reduction in the number of released virus particles but not in the number of assembling virions upon CD81 knockdown suggests that CD81 plays a role in a late stage of the virus budding process, likely at the final scission step. CD81 may facilitate viral scission by directly participating in the scission process, by recruiting host or viral scission proteins, or by organizing the lipid domains and making it conducive to viral scission.

### Distribution of CD81 in budding filamentous Udorn virus

**Figure 7:**
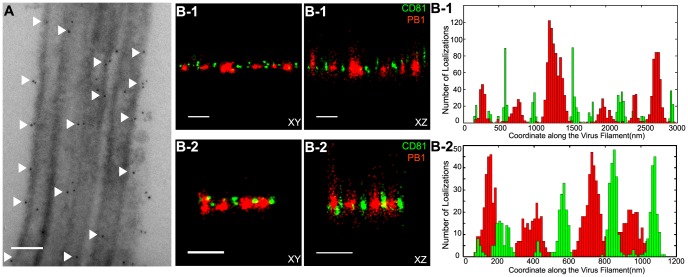
Scattered distribution of CD81 along budding filamentous Udorn virus. A) CD81 localizes along the filament of budding Udorn viruses. A549 cells were infected with Udorn virus for 18 hours and CD81 was immunogold labeled for electron microscopy. Shown here is a bundle of virus filament budding from the cell (the cell is not shown in order to magnify the virus filaments). Scale bar: 200 nm. B) CD81 and viral PB1 proteins appear to take an alternating distribution along filamentous Udorn virus. A549 cells were infected with Udorn virus for 16 hours and immunostained with anti-CD81 (green) and anti-PB1 (red) antibodies. CD81 was further probed with Alexa Fluor 405/Alexa Fluor 647-conjugated secondary antibody while PB1 was labeled with Atto 488 conjugated antibody for two-color 3D STORM imaging. Two example filamentous viruses were shown in B-1) and B-2). Left: xy projection images. Middle) xz projection images. Right) Localization distribution of CD81 and PB1 along the filament long axis. Scale bar: 500 nm.

Unlike WSN and X-31 strain, Udorn virus infection typically produces filaments that can reach 2 to 20 µm long [11], [35], [45], [46]. We visualized immunogold-labeled CD81 distribution in A549 cells infected by Udorn virus with electron microscopy and observed CD81 clusters in budding viral filaments. Notably, CD81 appeared to be distributed along the entire Udorn filament (Figure 7A).

As an alternative approach, we used a super-resolution fluorescence imaging technique, Stochastic Optical Reconstruction Microscopy (STORM), to measure the distribution of CD81 on the budding filament. STORM overcomes the diffraction limit of light microscopy by sequentially activating, imaging and localizing individual fluorescent photoswitchable molecules at high precision, thereby reconstituting images from the molecular localizations with nano-meter scale resolution [47], [48]. Here, we used a single-objective detection geometry and photoswitchable Alexa Fluor 647 and Atto 488 dyes to obtain a lateral resolution of 20–30 nm and axial resolution of 50–100 nm [48], [49]. To visualize the localization of CD81 in filamentous Udorn, we immunostained CD81 and viral PB1, and performed two-color 3D STORM imaging. Consistent with the results from electron microscopy (Figure 7A), we found that CD81 formed small clusters evenly distributed along the entire filament (Figure 7B). Adjacent CD81 clusters were usually separated by about 150∼200 nm. Curiously, CD81 appeared to be enriched between clusters of viral PB1 proteins (Figure 7B), but the significance of this alternating pattern is unclear.

## Discussion

CD81, a cellular tetraspannin protein, is critical for influenza viral infection [15], [16]. The infectivity of various strains of influenza viruses is strongly inhibited when cellular CD81 is depleted (Figure 1). In this work, we dissected the roles of CD81 on individual steps along the infection pathway from virus entry to egress. We found that CD81 plays functional roles in two separate steps of viral infection: viral fusion and virus budding.

### The role of CD81 in viral entry

The specific role of CD81 during influenza viral entry was determined using a series of independent assays. First, knocking down CD81 by siRNA led to ∼50% decrease in the percent of infected cells expressing viral proteins. The defect was not due to direct regulation of viral protein expression by CD81, since viral protein expression remained unchanged upon CD81 knockdown when influenza infection was induced by the acid-bypass treatment (Figure 1 and Figure 4). These results suggest that CD81 mediates influenza virus entry prior to viral gene expression. Next, CD81 knockdown did not affect virus binding, internalization or trafficking to early endosomes (Figure 2), but led to a significant defect in viral fusion (Figure 3). Furthermore, single-virus tracking experiments showed that half of internalized virus particles were trafficked into CD81-positive endosomes and underwent viral fusion within these endosomes, whereas the remaining half fused in CD81-negative endosomes (Figure 3). Notably, the fraction of viral fusion events occurring within CD81-positive endosomes correlated well with the 50% reduction in the percent of infected cells expressing viral proteins upon depletion of CD81, suggesting a role of CD81 in productive viral uncoating. Altogether, these results indicate that CD81 plays a role in influenza viral fusion. CD81 marks an endosomal route for productive virus uncoating process, though a parallel CD81-independent route also exists.

Interestingly, the role of CD81 in influenza virus entry appears to differ from the role of CD81 previously observed in HCV and HIV entry. As an essential co-receptor for HCV, CD81 is important for the endocytosis of HCV [27], [28], [50], [51]. Furthermore, CD81 interacts with HCV glycoprotein E2 and helps prime its fusogenic activity for low-pH dependent viral fusion [26], [30]. Moreover, CD81 negatively regulates HIV-cell fusion [52]. Incorporation of CD81 and CD81-associated tetraspanins suppresses the HIV-mediated cellular fusion processes [52], [53]. On the other hand, CD81 unlikely functions as a co-receptor or attachment factor for influenza viruses because the internalization of influenza viruses into cells does not require CD81. Influenza viral fusion does not need to be primed by CD81 either, as acid treatment is sufficient to trigger viral uncoating at the plasma membrane in CD81-depleted cells. Instead, our results suggest a role of CD81 in facilitating influenza virus fusion in endosomal compartments. Given that CD81 and CD81-associating proteins can organize membrane domains [17]–[20], CD81 may help organize the endosomal membrane for assisting influenza viral fusion. Alternatively, CD81 may play a role in trafficking influenza to fusion competent endosomal compartments. CD81 is highly enriched in multivesicular bodies (MVBs), an intermediate endosomal organelle on the maturation pathway of late endosomes and lysosomes [54]. CD81 depletion may inhibit the maturation of endosomes and thus compromise influenza virus fusion with endosomes.

### The role of CD81 in viral assembly

In addition to its role in virus uncoating, CD81 also plays a functional role in a later stage of influenza infection post viral fusion. The requirement of CD81 in a post-fusion stage was evident from the finding that CD81 depletion led to a significant decrease in virus titer even when the acid-bypass treatment was used to induce viral uncoating at the plasma membrane, thereby eliminating entry defects (Figure 1F). The decrease did not result from a defect in expression of viral proteins or trafficking of viral proteins to the plasma membrane (Figure 4), suggesting that the perturbation likely occurred at the virus assembly stage. Furthermore, the average number of budding virions attached to each infected cell did not change upon CD81 knockdown, whereas the number of virus particles released into the supernatant markedly decreased (Figure 6A, B). These results further narrowed the involvement of CD81 to a relatively late stage of the budding process, likely the scission step that severs the virus particle from the host cells. Supporting this notion, CD81 was specifically recruited to viral budding sites (Figure 5), and among the viral proteins, HA is likely important for recruiting CD81 to the virus budding sites (Figure S7). Interestingly, CD81 was specifically enriched at the tip and budding neck of the spherical and slightly elongated viruses (Figure 6C–E). Upon CD81 knockdown, the budding spherical viruses exhibited a consistent change in morphology: the budding virions appeared substantially elongated compared to their counterparts in control cells and failed to detach from the plasma membrane. Many budding viruses did not have their budding neck closed, indicating a defect in the final scission process (Figure 6F–H). Taken together, our observations indicate a role of CD81 in scission process that severs the budding virions from the plasma membrane. CD81 could be directly participating in the scission process, recruiting other host or viral scission proteins for this purpose, or organizing the membrane domain at the budding site and making it conducive for viral scission.

It is interesting to compare the role of CD81 in the assembly of influenza virus with that of other viruses. Previous studies have shown that HIV envelope proteins associate with a few tetraspanins, including CD81, and that HIV buds from the tetraspanin-enriched microdomains [55]–[57]. However, the exact role of CD81 in HIV egress remains unclear [53], [58]–[61]. One study reports that HIV infection is significantly impaired upon CD81 depletion or treatment with anti-CD81 antibodies [61], whereas two other papers report that depletion of tetraspanins does not affect the efficiency of HIV release whereas overexpression of tetraspanins results in decreased infectivity in released virions [53], [59]. Tetraspanins have also been proposed to facilitate cell-to-cell transmission of HTLV-1 infection [25]. The role of CD81 in the egress of influenza virus appears different from these previously reported roles of tetraspanins in HIV and HTLV-1 infection in that CD81 positively regulate viral scission.

It has been previously shown that influenza virus scission is dependent on viral M2 protein [62]. During virus budding, M2 is localized at the neck of budding viruses and mutation of its amphiphilic tail at the C-terminus leads to a marked defect in virus budding [62]. Interestingly, M2 is known to localize at the interface between lipid rafts and non-rafts region, while CD81 is partitioned into tetraspanin-enriched microdomains, a platform that resembles lipid rafts [18]. Thus, it is possible that CD81 facilitates the recruitment of M2 to the budding neck of the viruses. Future studies on the interaction between CD81 and M2 during the viral scission process would be of interest to further elucidate the mechanistic role of CD81. CD81 associates with tetraspanins and other tetraspanin-interacting proteins to form tetraspanin-enriched microdomains on cellular membranes [18], [20]. CD9, a tetraspanin that interacts with CD81, was previously identified in the purified virus particles [32]. Our preliminary results revealed that other tetraspanin family proteins were also incorporated into budding viruses (data not shown). Whether and how different components within the tetraspanin-enriched microdomains cooperate with each other in facilitating influenza virus budding remains an interesting question for future investigations.

## Materials and Methods

### Cell culture, viruses and reagents

A549 lung carcinoma cells (ATCC) were cultured in high glucose Dulbecco's modified Eagle medium (DMEM; Invitrogen) containing 10% fetal bovine serum (Serum International), and antibiotics (ATCC; 25 U/ml penicillin and 25 µg/ml streptomycin), and maintained in humidified, 5% CO_2_ environment at 37°C. For siRNA knockdown experiments, A549 cells were electroporated with 100 pmol siRNA constructs using program X-001 of Amaxa Lonza Nucleofector with Kit-T (Lonza, VVCA-1002). Experiments were performed 48 hours post electroporation. siGENOME non-targeting siRNA #1(Thermo Scientific) was used as a control siRNA. Six CD81 siRNA constructs were designed with the following sequences: CD81 siRNA 1: CACCU UCUAU GUAGG CAUCU A dTdT(Thermo Scientific); CD81 siRNA 2: AAGGA ACAUC AGGCA UGCUA A dTdT(Thermo Scientific); CD81 siRNA 3: GGAAC AUCAG GCAUG CUAATT (Qiagen); CD81 siRNA 4: CCUUC UAUGU AGGCA UCUATT(Qiagen); CD81 siRNA 5: GCCCA ACACC UUCUA UGUATT (Ambion); CD81 siRNA 6: CCACC UCAGU GCUCA AGAATT (Ambion). Note that CD81 siRNA 1 and CD81 siRNA 2 have been confirmed previously not to cause interferon-induced response [16]. For plasmid expression, 2 µg plasmids were electroporated with a similar procedure. The following plasmids were used in this study: CD81-mEmerald (human tetraspanin CD81 was cloned into the C terminal of mEmerald, with a 10 amino acid linker between mEmerald and CD81), RFP-Rab5 (gift from Professor Ari Helenius, Addgene, 14437 [63]), EYFP-Rab7 [41]), pCAGGS-HA/Ud and pCAGGS-NA/Ud (gifts from Professor Michael Farzan, Scripps Institute, FL).

The following viruses were used in this study: influenza virus X-31 was purchased from Charles River Laboratories; WSN and Udorn virus strains were gifts from Professor Robert Lamb (Northwestern University, Evanston, IL). Respiratory syncytial virus was purchased from Virapur. Pseudo-typed MLV virus was a gift from Professor Nir Hacohen (Broad Institute, Cambridge, MA).

The following primary antibodies were used in this study: mouse anti-CD81 antibody (BD Biosciences, 555675), FITC-conjugated anti-CD81 antibody (BD Bioscience, 551108), mouse anti-EEA1 (BD Biosciences, 610457), rabbit anti-EEA1 (Cell signaling, 3288s), rabbit anti-CD82 (Santa Cruz, c-16, SC-1087), mouse anti-CD63 (Abcam, ab8219), rabbit anti-EWIF (Fitzgerald, 70R-13159), mouse anti-ITGB1 (Millipore, MAB2253), mouse anti-tubulin (Sigma, T5076), mouse anti-EGFR (BD bioscience, 610016), rabbit anti-actin(Abcam, ab8227), rabbit anti-CD9 (Santa Cruz, H-110, sc-9148), goat anti-Udorn serum (gift from Professor Robert Lamb (Northwestern University, Evanston, IL)), mouse anti-M1 antibody (AbD Serotec, MCA401), goat anti-M1 antibody (Abcam, ab20910), mouse anti-influenza virus M2 antibody [14C2] (Abcam, ab5416), mouse anti-influenza virus NP antibody [AA5H] (Abcam, ab20343), mouse anti-Alexa Fluor 647 (Sigma, C1117), mouse anti-RSV fusion protein (AbD serotec, MCA490), goat anti-influenza virus PB1 antibody (Santa Cruz, vK-20), mouse anti-influenza virus HA antibody (Lifespan, LS-C58889), rabbit anti-influenza virus NA (gift from Professor Gillian Air (University of Oklahoma, Oklahoma, OK)).

The following secondary antibodies were used for immunofluorescence with conventional light microscopy or immunogold electron microscopy: Alexa Fluor 647 donkey anti-mouse (Jackson ImmunoResearch, 715-605-150), Cy3 donkey anti-mouse (Jackson ImmunoResearch, 715-165-150), Alexa Fluor 488 donkey anti-mouse (Jackson ImmunoResearch, 715-545-150), Alexa Fluor 488 donkey anti-rabbit (Jackson ImmunoResearch, 711-545-152), Cy3 donkey anti-rabbit (Jackson ImmunoResearch, 711-165-152), Alexa Fluor 647 bovine anti-goat (Jackson ImmunoResearch, 805-605-180), Alexa Fluor 488 bovine anti-goat (Jackson ImmunoResearch,805-545-180), 6 nm gold conjugated goat anti-mouse IgG (Jackson ImmunoResearch, 115-195-146).

The following secondary antibodies were used for immunofluoresence with STORM: donkey anti-mouse (Jackson ImmunoResearch, 715-005-150) labeled with Atto 488 or Alexa Fluor 405 and Alexa Fluor 647, Bovine anti-goat (Jackson ImmunoResearch, 805-005-180) labeled with Atto 488 or Alexa Fluor 405 and Alexa Fluor 647. To label antibodies with Alexa Fluor 405 and Alexa Fluor 647, 80 µl antibody (1.3 mg/ml) were mixed with 10 µl 1M NaHCO_3_, 8 µg Alexa Fluor 405 and 1.2 µg Alexa Fluor 647 dissolved in DMSO for 30 minutes. To label antibodies with Atto 488, the conditions were similar except 1.6 µg Atto 488 was used for the labeling reaction. The mixture was then filtered through a NAP-5 gel filtration column (GE Healthcare) to collect labeled antibody. Atto 488 emitted about 1100∼1300 photons per switching cycle, which is significantly lower than that of Alexa Fluor 647 (4000∼5000 photons per switching cycle) [49].

### Virus infection

For virus infection, A549 cells were first inoculated with different doses of viruses diluted in DMEM (without serum) for 90 minutes at 37°C. Cells were washed with PBS twice to remove unbound viruses, and subsequently incubated with pre-warmed full DMEM medium and maintained at 37°C. For measuring the influenza virus titer (X-31, WSN, and Udorn) without using the acid-bypass treatment, a total of 36 hours were allowed for virus infection before collecting the supernatant for the plaque assay as described below. For RSV virus infection, A549 cells were infected with RSV for 24 hours, followed by immunostaining with anti-fusion protein (F protein) antibodies and analysis by flow cytometry. For pseudo-typed MLV virus infection, a 24-hour was allowed for infection and cells were directly fixed to assay the GFP fluorescent intensity by flow cytometry.

To ensure equal amounts of viral entry in control and CD81 siRNA treated cells for post-entry studies, an acid-bypass treatment was conducted to induce viral fusion at the plasma membrane. siRNA treated A549 cells were allowed to bind with influenza virus at 4°C for one hour. After extensive washes with cold PBS, a pre-warmed low pH buffer (PBS, pH 4.5) was added in for two minutes. The low pH buffer was then neutralized with culture medium and cells were placed with pre-warmed fresh culture medium afterwards. For measuring the influenza virus titer (X-31 and WSN) with the acid-bypass treatment, a total of 17 hours were allowed for virus infection before collecting the supernatant for the plaque assay.

### Plaque assay

Cells were infected with influenza virus for indicated amounts of time as described above and supernatant was collected to assay the virus titer at the end of each time point. Serial dilutions of the supernatant were used to inoculate MDCK-Texas cells (Kind gift from Robert Lamb) seeded in 6-well plates for 90 minutes at 37°C. After washing with PBS twice, a 3 ml agar overlay of DMEM containing 30% Noble agar (Affymetrix), antibiotics and 2 µg/ml acetylated trypsin (Sigma) was placed on cells. The plates were incubated at 37°C. After about two days, the agar disks were removed carefully and cells were immediately stained with crystal violet solution (1∶1,000 V/W crystal violet, 30% ethanol in water) for 10 to 15 minutes, which allowed for an easy quantification of the number of plaques. Virus titer (PFU/mL) = number of plaques/(dilution factor×inoculation volume (mL)). For each condition, samples were tested with triplicates.

### Influenza virus labeling

Influenza virus X-31 was either labeled with lipophilic dye DiD (Invitrogen, D7757) or Alexa Fluor 647 (Invitrogen, A-20006) as previously described [42]. For the labeling reaction, 100 µl of the original virus stock (2 mg/ml protein concentration) was incubated with either 3 µl of 25 mM DiD or 3 µg Alexa Fluor 647 dissolved in DMSO for two hours or one hour respectively with gentle vortexing in the dark at room temperature. Unincorporated dye was removed by buffer exchange into the Hepes 145 buffer (50 mM Hepes, pH 7.4, 145 mM NaCl) by using NAP-5 gel filtration columns (GE Healthcare). The labeled virus was aliquoted, snap-frozen in liquid nitrogen, and stored at −80°C. Immediately before experiments, the labeled virus was thawed and filtered through a 0.2 µm pore size syringe filter (Supor membrane, Pall) to remove viral aggregates. Labeled viruses are infectious, as confirmed with standard plaque assays (data not shown).

### Virus binding assay

Control or CD81-knockdown A549 cells were allowed to bind with DiD-labeled X-31 diluted in DMEM (without serum) for 30 minutes at 4°C. After extensive washes with cold PBS to remove unbound viruses, cells were trypsinized and immediately fixed with 2% paraformaldehyde (PFA) for 20 minutes at room temperature. After washing PFA away with PBS, the DiD fluorescent intensity was measured by a flow cytometer (BD bioscience). At least 10,000 cells were quantified for each measurement. The data was analyzed via FlowJo.

### Virus internalization assay

Control or CD81-knockdown A549 cells were allowed to bind with 3×10^4^ PFU/ml Alexa Fluor 647-labeled X-31 virus diluted in DMEM (without serum) on ice for 30 minutes at 4°C. After extensive washes with cold PBS to remove unbound viruses, pre-warmed full culture medium was added in and the virus was allowed to internalize at 37°C for indicated amounts of time. At the end of each time point, cells were washed with PBS, directly fixed with 4% PFA for 20 minutes at room temperature. In order to distinguish the surface-bound versus internalized virus particles, a non-permeablizing immunofluorescence in the absence of detergents was performed by using a mouse anti-Alexa Fluor 647 primary antibody (Sigma, C1117), followed by staining with an Alexa Fluor 555-conjugated donkey anti-mouse secondary antibody (Invitrogen, A31570). The samples were imaged using a custom-built spinning disk confocal microscope. Non-internalized virus particles were stained with both Alexa Fluor 647 and Alexa Fluor 555, while the internalized virus particles—inaccessible to the antibodies—exhibited only the Alexa Fluor 647 signal. To quantify the number of particles internalized per cell, we used the maximum z-projection of the confocal z-stacks, and counted both the total number of virus particles (with Alexa Fluor647 signal) and the number of non-internalized virus particles (with both Alexa Fluor 647 and Alexa Fluor555 signals), and the number of internalized particles (with Alexa Fluor 647 but not Alexa Fluor 555 signal). A low enough number of virus particles (∼15 particles) was internalized each cell to minimize the possibility of multiple virus particles sorting into the same vesicle. Statistical analysis was performed using a two-tailed student t-test.

### Western blotting

Cell lysate samples were prepared with Laemmli sample buffer (Bio-Rad, 161-037) and run on a 4–15% Tris-HCL polyacrylamide gel (Bio-Rad). After transferring the protein onto Hybond polyvinylidene difluoride membranes (GE Healthcare), the membrane was blocked with 5% nonfat-milk in TBS-Tween for 1 hour, followed with incubation of primary antibody overnight at 4°C, a three 10 minutes wash step with TBS-Tween, and a one hour incubation of HRP-conjugated secondary antibody at room temperature. The signal was detected with TMA-6 (TMA-100, Lumigen) and developed to Kodak films. Note that CD81 and CD63 could only be detected under non-reducing conditions.

### Flow cytometry

For flow cytometry analysis, the procedures were similar to what was previously described [64]. Briefly, for measuring total protein expression level (CD81 or viral proteins), cells were collected and fixed with 2% PFA for 20 minutes. After washing with PBS once, cells were permeablized with buffer P (0.075% Saponin, 10% BSA in PBS) for 20 minutes at room temperature. Cells were incubated with primary antibodies diluted in buffer P (1∶1,000) for 1 hour and washed with buffer P three times before incubating with secondary antibodies for another 45 minutes. Secondary antibodies were also diluted with buffer P (1∶1,000). After washing with buffer P, cells were resuspended with PBS and then analyzed by flow cytometry. The data was analyzed by FlowJo. Cells were gated based on FSC and SSC scattering, and a histogram was generated based on the fluorescence intensity profile. For cells that were infected with influenza virus, a second gate was set based on comparison of fluorescence intensity of uninfected versus infected cells. The population that falls into the second gate corresponds to the percent of infected cells in each sample, from which the mean fluorescence intensity was analyzed to infer the viral protein expression level. To probe surface protein expression, all steps were similar except detergent was excluded.

### Immunofluorescence

For imaging-based experiments, A549 cells seeded in Lab-Tek 8 well glass dishes were fixed with 4% PFA for 20 minutes at room temperature. Unless specified, fixed cells were permeablized with 0.1% Triton-X100 in PBS for 5 minutes, washed with PBS twice and incubated with blocking buffer PBSA (3% BSA in PBS, or 5% bovine serum in PBS) for 30 minutes. Cells were then incubated with primary antibodies diluted in PBSA (1∶500) for 1 hour. Followed by three PBS washes (5 minutes each), secondary antibodies were added for another 1 hour. Afterwards, cells were washed with PBS for three times again before imaging. For STORM, a post-fixation step was followed with 3% PFA and 0.1% glutaraldehyde (GA) in PBS for 20 minutes. For immunostaining surface protein only, permeablization was not performed after fixation. When antibody species conflict existed, labeled primary antibodies (CD81-FITC, BD Bioscience; HA-Alexa Fluor 647) were used as needed.

To quantify the colocalization ratio between internalized virus and cellular proteins (EEA1 and CD81), samples were prepared similarly as in virus internalization assay. After probing the surface-bound virus particles with anti-Alexa Fluor 647 antibody, cells were permeabilized with 0.1% Triton-X100 in PBS, and a subsequent indirect immunofluorescence was performed to stain against each protein. Images were acquired by confocal microscopy and at least 40∼100 randomly chosen cells were analyzed manually for each condition. Only internalized virus particles were used to quantify the fraction of viruses colocalized with CD81 or EEA1.

### ELISA

Cells were infected with influenza virus with acid bypass for 17 hours and the supernatant was collected to assay the total amount of viral protein in the released virus particles. M1 was chosen due to its abundance in the virus to maximize signal. Nunc 96 well plates (eBoscience, 44-2404-21) were incubated with capture antibody (Goat anti-M1, Abcam, 1∶1000 diluted in 0.2 M sodium carbonate/bicarbonate buffer, pH 9.4) at room temperature for 2 hours. After three 5 minutes wash with PBST (0.05% Tween in PBS), the plates were blocked with PBSA (2% BSA in PBST) for 1 hour. The supernatant was mixed with RIPA buffer (1∶2 dilution), and added in each well for overnight incubation at 4°C. The samples were washed three times with PBST, and incubated with detection antibody (Mouse anti-M1, AbD Serotec, 1∶600 dilution in PBSA) for 1 hour at room temperature, washed three times, and further incubated with HRP-conjugated goat anti-Mouse antibody (Bio-Rad, 172-1011, 1∶5,000) for 1 hour. TMB substrate (Thermo Scientific, N301) was used to detect HRP activity, and the reaction was stopped by 0.18 M sulfuric acid before measuring the absorbance at 450 nm. The experiment was performed with triplicate samples from independent infections, with three measurements for each sample. To confirm the efficiency of detection, purified X-31 virus (Charles River laboratory) was used as a standard sample (data not shown).

### Quantification of the number of released virions

Control and CD81 siRNA treated cells were infected with influenza virus by acid bypass treatment. At 17 hours post infection, supernatant was collected to assay for the number of released virions through immunofluorescence. Briefly, the supernatant was absorbed on poly-lysine coated Lab-Tek 8 well glass dishes at 4°C overnight. After washing away unbound virions with PBS twice, the sample was fixed with 4% PFA for 15 minutes, blocked with 3% PBSA buffer, immunostained with anti-HA and anti-M1 antibody, and then imaged by confocal microscopy. More than 150 randomly selected regions were imaged, and the number of particles positive for HA and M1 staining was quantified (with more than 600 virus particles).

### Single particle tracking

The single-particle tracking experiment has been described in detail previously [5], [41]–[43]. Briefly, A549 cells were nucleofected with CD81-mEmerald or RFP-Rab5 plasmids 24 hours prior to single virus tracking experiments. After washing the cells with pre-warmed PBS twice, 2.6×10^4^ PFU/ml DiD-labeled X-31 virus diluted in imaging buffer (9 parts DMEM without phenol red, 1 part pH 8 Hepes buffer, supplemented with oxygen scavenge system: 0.8 mg/ml dihydroxybenzoic acid (PCA, Sigma, 37580), 0.5 U/ml protocatechuate 3,4- dioxygenase (PCD, Sigma, P8279)) was added to the cells. The objective and stage of the microscope were heated to maintain the temperature at 37°C for the cells. Image acquisition began immediately after adding DiD-labeled virus *in situ*. To obtain a simultaneous imaging of DiD-labeled virus and cellular protein, DiD was excited with a 647 nm krypton laser (Coherent) while mEmerald and RFP was excited with a 488 nm argon ion laser (Coherent) and a 561 nm solid state laser (CrystaLaser) respectively. Fluorescence emissions from DiD and mEmerald/RFP were separated by a 630 nm long-pass dichroic, filtered with bandpass filters (705/40 for 647 channel, 525/40 for 488 channel, and 605/70 for 561 channel) and imaged on a EMCCD camera (Andor) with 0.5 second exposure time. The imaging analysis was performed as described previously [5]. Briefly, for each image, the fluorescence signal collected from the DiD channel was convolved with a Gaussian spatial filter to remove background and noise. To identify the virus peaks, the algorithm performs recursive integration over bright regions connected to each local maximum. The centroid of each fluorescent peak was computed to determine the virus particle position, and the trajectories were obtained by reconstructing paired peaks between adjacent frames with similar proximity and intensity. The fluorescence intensity of DiD was plotted versus time.

### Bulk viral fusion assay

Control or CD81-knockdown A549 cells seeded in Lab-Tek 8 well glass dishes were rinsed with cold PBS first, and then incubated with 2×10^4^ PFU/ml DiD-labeled X-31 diluted in DMEM (without serum) for 45 minutes at 4°C. After washing away unbound viruses with cold PBS twice, pre-warmed full culture medium was added in and cells were maintained at 37°C for indicated amounts of time. At the end of each time point, cells were trypsinized and immediately fixed with 2% PFA for 20 minutes. Afterwards, cells were washed and resuspended with PBS. DiD-fluorescent intensity was measured through a flow cytometer. At least 10,000 cells were measured for each measurement with duplicates for each condition. The data was analyzed with FlowJo. The normalized viral fusion extent (mean DiD intensity at each time point-initial mean DiD intensity)/Initial mean DiD intensity) was plotted.

### STORM

STORM experiments were performed as previously described on an Olympus IX71 inverted optical microscope [65]. Three lasers were used in this study for STORM: 657 nm (RCL-300-656; Crystalaser), 488 nm (Sapphire 460-10; Coherent) and 405 nm (CUBE 405-50C; Coherent). A high-numerical-aperture (NA) oil-immersion objective (100× UPlanSapo, NA1.4; Olympus) was used to collect the fluorescence emission, which is imaged onto a back-illuminated electron-multiplying charge-coupled device (EMCCD) camera (iXON DU-897; Andor). For two-color 3D imaging of Alexa Fluor 647 and Atto 488, two imaging laser beams (488 nm and 657 nm) and an activation laser beam (405 nm) were reflected by a custom-designed polychroic mirror (z488/647/780rpc; Chroma). Fluorescence emission from Alexa Fluor 647 and Atto 488 were separated by a 630 nm long-pass dichroic mounted on a commercial beamsplitting device (3D Dual-View with a cylindrical lens, 100 cm focal length; Photometrics). Two bandpass filters: FF01-535/50(Semrock) and ET705/72m (Chroma) were used to filter for the short-wavelength and long-wavelength channel independently. In addition to the bandpass filters, a double-notch filter (NF01-488/647; Semrock) was added before the Dual-View (Photometrics) to block the two excitation laser beams. STORM imaging for each channel was performed at 60 Hz sequentially and each channel was imaged onto 256×256 pixels in the EMCCD camera (iXON DU-897). STORM images were generated using similar methods as previously described [65]. The STORM images in the Alexa Fluor 647 and Atto 488 channels were aligned by a third-order polynomial warping map in three dimensions obtained from calibration images of 100-nm Tetraspeck fluorescent beads. The residual alignment error was ∼7 nm in x-y and ∼20 nm in z dimensions. To correct for the sample drift during imaging acquisition, we relied on the correlation function of imaging itself to correct for the lateral and axial drift, as previously described [48]. Since the imaging acquisition was performed sequentially with the longer wavelength channel first, 647 channel was drift-corrected to the last frame of the 647 nm acquisition while 488 channel was drift-corrected to the first frame of the 488 nm acquisition. The spatial resolution measured was 20∼30 nm laterally and 50∼60 nm axially for Alexa Fluor 647; while Atto 488 gives a lateral resolution of 30∼40 nm and axial resolution of ∼100 nm.

### Electron microscopy

A549 cells were infected with influenza viruses at a MOI of 2. Infection was allowed to proceed for a total of 15 hours before fixing with 2.5% PFA/GA in 0.1M sodium cacodylate buffer, pH 7.4 (Electron Microscopy Sciences, 15949) at room temperature for at least 1 hour. The cells were post-fixed for 30 minutes in 1% Osmium tetroxide (OsO4)/1.5% potassium ferrocyanide (KFeCN6), washed in water three times and incubated in 1% aqueous uranyl acetate for 30 minutes followed by two washes in water and subsequent dehydration in grades of alcohol (5 minutes each; 50%, 70%, 95%, 2×100%). Cells were removed from the dish in propyleneoxide, pelleted at 3000 rpm for 3 minutes and infiltrated for 2 hours in a 1∶1 mixture of propyleneoxide and TAAB Epon (Marivac Canada Inc. St. Laurent, Canada). The samples subsequently embedded in TAAB Epon and polymerized at 60°C for 48 hours. Ultrathin sections (about 60 nm) were cut on a Reichert Ultracut-S microtome, transferred onto copper grids stained with lead citrate and examined in a TecnaiG^2^ Spirit BioTWIN and images were recorded with an AMT 2k CCD camera.

For immunogold electron microscopy, at the end of indicated time points of virus infection, cells were rinsed with PBS once, fixed with 4% PFA for 15 minutes, and blocked with PBSA (3% BSA in PBS) for 30 minutes. Primary antibodies diluted in PBSA (1∶500) were incubated with cells for overnight at 4°C. After three washes with PBS, cells were treated with 6 nm gold-conjugated secondary antibodies (1∶40) for four hours, post-fixed with 2.5% PFA/GA in 0.1 M sodium cacodylate buffer for at least one hour. The sample were embedded and sectioned as described above for transmission electron microscope imaging.

## Supporting Information

Figure S1 **CD81 depletion efficiency with different siRNA constructs.** A) Six different siRNA constructs were tested for CD81-knockdown efficiency after 48 hours of electroporation. CD81 expression was measured by flow cytometry following staining with anti-CD81 primary antibody and fluorescently labeled secondary antibody. At least 10,000 cells were measured for each condition. The orange, blue and magenta curves correspond to the intensity profiles measured for cells without addition of the primary antibody, control (non-targeting) siRNA treated cells immunostained for CD81, and CD81 siRNA treated cells immunostained for CD81, respectively. B) Quantitative summary of the measurements from (A). CD81 siRNA 1 was selected for subsequent studies. C) A549 cells were treated with control or CD81 siRNA 1 for 48 hours. Cells were either permeabilized with detergent or not permeabilized to detect the total amount of CD81 in cells or the amount of CD81 on the cell surface, respectively. The intracellular CD81 level was then calculated by subtracting the amount of CD81 on the cell surface from the total amount of CD81. Based on the quantification, the cell-surface and intracellular CD81 fractions were ∼92% and 8% in control cells, respectively. The partition is similar in CD81-knockdown cells (87% versus 13% for cell-surface and intracellular CD81, respectively). A two-tailed *t-test* was performed in each case and the p values were provided. D) CD81 knockdown does not affect the expression of CD81-associating proteins. A549 cells were treated with control or CD81 siRNA for 48 hours. Cells were harvested for western blotting with indicated antibodies. Actin and tubulin were used as loading controls.
(EPS)

Figure S2 **The CD81-mEmerald expression does not affect CD81 distribution or influenza viral fusion and infection.** A) A549 cells were nucleofected with CD81-mEmerald plasmid for 24 hours, and subsequently immunostained with anti-CD81 antibody. Note that there is an almost complete overlap between CD81-mEmerald signal and CD81 antibody staining. B) CD81 partially colocalizes with early endosomes marker. A549 cells were nucleofected with CD81-mEmerald plasmid for 24 hours, and subsequently immunostained with anti-EEA1 antibody. C) A549 cells were immunostained with anti-CD81 and anti-EEA1 antibody to show the partial colocalization between endogenous CD81 and EEA1. D) Quantification of colocalization between CD81 and EEA1. We randomly selected 2500∼3000 CD81+ vesicles from confocal slices, and quantified the colocalization between CD81-mEmerald or endogenous CD81 and EEA1. For the CD81-mEmerald expressing cells, about 37±11% of the CD81-mEmerald+ vesicles were positive for EEA1 and about 33±12% of the EEA1+ vesicles were positive for CD81-mEmerald. A similar colocalization level was observed for endogenous CD81 and EEA1 in untransfected cells: about 34±13% of the CD81+ vesicles were positive for EEA1 and about 38±14% of the EEA1+ vesicles were positive for CD81. E) CD81-mEmerald expression does not affect influenza viral fusion. A549 cells were nucleofected with CD81-mEmerald or control GFP plasmid for 24 hours. Bulk viral fusion assay was performed at indicated times. The error bar was standard deviation from triplicates. F) CD81-mEmerald expression does not affect influenza infection. A549 cells were nucleofected with CD81-mEmerald or control GFP plasmid for 24 hours. Cells were infected with X-31 or WSN with a MOI<0.1 for 36 hours. The virus titer in the supernatant was measured by plaque assays. The error bar was standard deviation from triplicates.
(EPS)

Figure S3 **Fusion of influenza viruses primarily occurs in Rab5-positive endosomes in both control and CD81-knockdown cells.** A) An influenza virus particle enters and fuses within a Rab5-positive endosome after entry. Live-cell confocal imaging of DiD-labeled X-31 added *in situ* to RFP-Rab5 expressing A549 cells maintained at 37°C. The images were collected with a 0.5 s interval. A-1) Several snapshots taken at different time points with the virus indicated by the white circles. A-2) The fluorescence signal of the indicated DiD-labeled virus as a function of time. Note that viral fusion event occurred at 434 s. B) Influenza virus occasionally fuses in a Rab5-negative endosome. B-1) Several snapshots taken at different time points with the virus indicated by the white circles. B-2) The fluorescence signal of the indicated DiD labeled virus as a function of time. Viral fusion event occurred at 85 s. C) C-1) Among 40 virus particles that fused in control cells, 86±4% enter and fuse within Rab5+ endosomes, whereas the remaining fuse in Rab5- endosomes. C-2) Among 35 virus particles that fused in CD81-knockdown cells, 89±3% enter and fuse within Rab5+ endosomes, whereas the remaining fuse in Rab5- endosomes. The error is the standard deviation from two independent experiments. D) Virus fusion in RFP-Rab5 expressing cells is impaired upon CD81 depletion. Experiments were performed similarly as in Figure 3F except that cells were nucleofected with RFP-Rab5 plasmids. Only those cells expressing RFP-Rab5 were selected for flow cytometry analysis. Error bars are standard deviation from triplicate measurements. A two-tailed *t-test* was performed and the p value is provided.
(EPS)

Figure S4 **Colocalization between CD81 and various viral proteins at the virus budding sites.** A) Cells were infected with X-31 for 16 hours, fixed, permeabilized and immunostained with anti-NA (red) and anti-CD81 (green). Scale bar: 10 µm. B) Cells were infected with X-31 for 16 hours, fixed and immunostained with anti-M2 (red) and anti-CD81 (green) without permeabilization. Scale bar: 10 µm. C) Cells were infected with Udorn for 16 hours, fixed, permeabilized and immunostained with anti-HA (red) and anti-PB1 (green). Scale bar: 10 µm. D) Cells were infected with Udorn for 16 hours, fixed, permeabilized and immunostained with anti-M2 (red) and anti-PB1 (green). Scale bar: 10 µm. E) Cells were infected with Udorn for 16 hours, fixed, permeabilized and immunostained with anti-NA (red) and anti-CD81 (green). Scale bar: 10 µm. F) Cells were infected with Udorn for 16 hours, fixed, permeabilized and immunostained with anti-HA (red) and anti-CD81 (green). Scale bar: 10 µm. G) Cells were infected with Udorn for 16 hours, fixed, permeabilized and immunostained with anti-Udorn serum (red) and anti-CD81 (green). Scale bar: 10 µm. H) CD81-knockdown cells were infected with Udorn for 16 hours, fixed, permeabilized and immunostained with anti-Udorn serum (red) and anti-CD81 (green). Scale bar: 10 µm.
(EPS)

Figure S5 **CD81 expression level is decreased modestly with influenza virus infection.** A) The amount of CD81 expression in uninfected or X-31 infected cells was assayed through flow cytometry. A549 cells were immunostained with NP and CD81. Cells were gated based on their NP intensity for further analysis of CD81 expression level. On the left panel, the black, red and blue curves correspond to the NP intensity profiles measured for cells without adding the primary antibody against NP, cells not infected with influenza virus and cells infected with influenza virus, respectively. Specifically, NP- cells that correspond to cells in the orange band in the left panel were selected for analyzing CD81 intensity in uninfected cells, while NP+ cells that correspond to cells in the blue band in the left panel were selected for analyzing CD81 intensity in X-31 infected cells. The fluorescence intensity of CD81 is plotted on the right panel, where the black, red and blue curves correspond to the CD81 intensity profiles measured for cells without adding the primary antibody against CD81, NP- cells and NP+ cells, respectively. At least 10, 000 cells were quantified in each condition. B) Quantification of CD81 expression level in cells with or without X-31 infection based on the analysis described in (A). The cells were infected by X-31 for 17 hours. Note that there is about 20% decrease in CD81 expression with X-31 infection. The error bars are the standard deviation from duplicate measurements.
(EPS)

Figure S6 **CD81 is enriched in the budding WSN virions and CD81-knockdown impairs scission of the X-31 virus.** A) CD81 is incorporated in budding WSN viruses. Cells were infected with WSN for 15 hours, and CD81 was immunogold labeled for electron microscopy. An enlarged image of the area in the white box is shown on the upper right. Scale bar: 100 nm. B) EM image of budding X-31 virus in control cells. A549 cells were infected with X-31 virus with the acid-bypass treatment for 16 hours. Scale bar: 200 nm. C) Budding X-31 viruses in CD81-knockdown cells. A549 cells were infected with X-31 virus with the acid-bypass treatment for 16 hours. Note that a substantial fraction of budding viruses have their open membrane neck connected to the plasma membrane (arrowheads). Scale bar: 200 nm.
(EPS)

Figure S7 **HA alone can recruit CD81 into HA-enriched clusters.** A) A549 cells were electroporated with pCAGGS-HA/Udorn plasmids for 24 hours and immunostained with HA (red) and CD81 (green) antibodies. HA (red) was probed with Alexa Fluor 647 labeled anti-HA and CD81 (green) was probed with FITC conjugated anti-CD81. Images are XY cross-sections from confocal images. Scale bar: 10 µm. B) Experiments were performed similarly as in (A) except the cells were mock transfected with a vector plasmid. Images are XY cross-sections from confocal images. Scale bar: 10 µm. C) A549 cells were electroporated with pCAGGS-NA/Ud plasmid for 24 hours, fixed and immunostained with anti-CD81 (green) and anti-NA (red) antibodies. NA (red) was probed with Alexa Fluor 647 labeled anti-NA and CD81 (green) was probed with FITC conjugated anti-CD81. Scale bar: 10 µm. D) Experiments were performed similarly as in (C) except the cells were mock transfected with a vector plasmid. Scale bar: 10 µm.
(EPS)

Movie S1 **An influenza virus particle enters and fuses within a CD81-positive endosome after entry.** Live cell confocal imaging of DiD-labeled X-31 added *in situ* to CD81-mEmearld expressing A549 cells maintained at 37°C. The images were collected with a 0.5 s interval. Movie is played seven times faster than the raw acquired data.
(WMV)

Movie S2 **An influenza virus particle fuses in a CD81-negative endosome.** Live cell confocal imaging of DiD-labeled X-31 added *in situ* to CD81-mEmearld expressing A549 cells maintained at 37°C. The images were collected with a 0.5 s interval. Movie is played seven times faster than the raw acquired data.
(WMV)
